# Clinical features of pachyvessels associated with polypoidal choroidal vasculopathy in chronic central serous chorioretinopathy

**DOI:** 10.1038/s41598-021-93476-2

**Published:** 2021-07-06

**Authors:** Te-An Wang, Wei-Chun Chan, Shawn H. Tsai, Lee-Jen Chen

**Affiliations:** 1 Department of Ophthalmology, Mackay Memorial Hospital, Address: No. 92, Sec. 2, Zhongshan N. Rd., Zhongshan Dist., Taipei City, 10449 Taiwan; 2 Department of Optometry, Mackay Junior College of Medicine, Nursing and Management, Taipei City, Taiwan

**Keywords:** Macular degeneration, Retinal diseases, Uveal diseases

## Abstract

To investigate the association between clinical features of chronic central serous chorioretinopathy (CSC) and subsequent development of polypoidal choroidal vasculopathy (PCV). Characteristics and treatment response of PCV secondary to CSC were described. This retrospective observational study included 18 patients with chronic CSC (18 eyes) with subsequent PCV and 36 controls (36 eyes) with chronic CSC without PCV development during follow-up. Clinical features were compared between the two groups. A logistic regression model was used to evaluate the risk factor of PCV formation. Treatments for PCV included anti-vascular endothelial growth factor (VEGF) monotherapy, photodynamic therapy (PDT), or PDT and anti-VEGF combination treatment. Subretinal fluid on optical coherence tomography images were assessed after treatments. Significant between-group differences were observed in best-corrected visual acuity after disease resolution and presence of pachyvessels (*P* = .001 and *P* = .003, respectively). The presence of pachyvessels in chronic CSC was associated with subsequent PCV (odds ratio = 6.00; 95% CI, 1.74–20.68; *P* = .005). CSC recurrence and subfoveal choroidal thickness (SFCT) were not significantly associated with subsequent PCV development (*P* = .393 and *P* = .911, respectively). The mean age of PCV diagnosis was 51 years, and the mean time from CSC diagnosis to PCV confirmation was 77.8 months. The mean (range) SFCT of PCV was 327.7 (134–599) μm. Nine patients received anti-VEGF monotherapy and 5 had disease remission. Four patients received PDT and anti-VEGF combination treatment and all of the 4 had disease remission. In chronic CSC, pachyvessel characteristics are associated with subsequent PCV development. This result will assist clinicians to evaluate CSC in clinical practice and provide insights into the pathogenesis of PCV.

## Introduction

Central serous chorioretinopathy (CSC) is among the most common vision-threatening diseases and is characterized by serous retinal detachments (SRDs) with or without focal pigment epithelial detachments (PEDs) and altered retinal pigment epithelium (RPE). An SRD often resolves within 3 to 4 months, but it recurs in up to 50% of cases^1^. CSC has a distinct disease pattern based on clinical courses. In acute CSC, an SRD resolves spontaneously in 4 to 6 months with a good visual prognosis. Persistent SRDs longer than 4 to 6 months and RPE atrophy characterize chronic CSC. RPE atrophy can cause vision reduction, and different pathognomonic patterns on fundus autofluorescence (FAF) image are utilized to evaluate disease progression^2,3^. In addition, chronic CSC can lead to chronic cystoid macular edema, intraretinal lipid deposition, and secondary choroidal neovascularization (CNV)^1^.

Pachychoroid is a novel term to describe a phenotype characterized by increased choroidal thickness which is attributable to the presence of pachyvessels, and focal choriocapillaris thinning. The pachychoroid concept recognizes that there might be a mechanistic relationship between CSC, its precursor, pachychoroid pigment epitheliopathy (PPE), and neovascular sequences such as pachychoroid neovasculopathy (PNV) and polypoidal choroidal vasculopathy (PCV), also recently referred to as aneurysmal type 1 neovascularization^4–6^. PCV is an exudative maculopathy, which is characterized by abnormality of inner choroidal vascular network and type 1 (sub-RPE) neovascularization^7^. The causes of PCV are incompletely understood. Studies have hypothesized that pachyvessels and alteration of the inner choroidal structure would be related to the pathogenesis of PCV^6,8^.

Choroidal pachyvessels, also known as dilated choroidal vessels in Haller’s layer, cause increased choroidal thickness and morphological alterations of the overlying choriocapillaris^9^. It is hypothesized that the presence of pachyvessels in CSC might be related to choriocapillaris atrophy and macular neovascularization^6^. However, no previous study had addressed the association between pachyvessel morphology in CSC and subsequent development of macular neovascularization. In this retrospective study, we evaluated the association between the clinical features of chronic CSC and subsequent development of PCV. We also described the characteristics, morphology, and treatment response of PCV secondary to CSC.

## Methods

This retrospective observational study was conducted at two tertiary referral hospitals: Mackay Memorial Hospital, Taipei branch, and Mackay Memorial Hospital, Tamsui branch. The study followed the tenets of the Declaration of Helsinki and was approved by the institutional review boards of Mackay Memorial Hospital. Informed consent forms were obtained from all patients to agree participation in the study.

All patients with a discharge diagnosis of CSC from January 2009 to November 2019 were eligible for inclusion in the study. The inclusion criteria were as follows: (1) having CSC proven by indocyanine green angiography (ICGA) or fluorescein angiography (FA); (2) having CSC with persistent SRD for more than 3 months and (3) having a minimum follow-up of 12 months and having complete medical records. The exclusion criteria were as follows: (1) being suspected of having AMD or PCV through FA or ICGA; (2) having coexisting retinal diseases including diabetic retinopathy, retinal venous or arterial occlusion, vitreoretinal diseases, ocular inflammation, or hereditary retinal diseases; (3) having high myopia (> − 6 diopters); (4) previously undergoing laser treatment, photodynamic therapy (PDT), or intravitreal anti-VEGF injection for the treatment of CSC; or (5) having poor image quality due to comorbid ocular diseases. The included patients were divided into two groups. When the diagnosis of CSC was established, SD-OCT, FA, and ICGA were applied to exclude the presence of CNV and PCV. Patients with subsequent development of PCV were assigned to the case (PCV) group. Patients without evidence of PCV during follow-up were assigned to the control group.

### Baseline and follow-up examination

At the initial visit, all patients received comprehensive ophthalmic examinations, including best-corrected visual acuity (BCVA) assessment (assessed using a Snellen chart), autorefraction (Auto-kerato-refractometer KR-800; Topcon, Tokyo, Japan), intraocular pressure measurement, slit-lamp examination, color fundus photography, spectral-domain optical coherence tomography (SD-OCT; CIRRUS HD-OCT model 4000 and model 5000; Carl Zeiss Meditec, Dublin, CA, and Spectralis; Heidelberg Engineering, Heidelberg, Germany), FA, and ICGA (HRA, Heidelberg Engineering). During the follow-up visit, ophthalmic examinations were performed, including BCVA, intraocular pressure measurement, slit-lamp examination, dilated fundus examination, color fundus photography, and SD-OCT.

**Figure 1:**
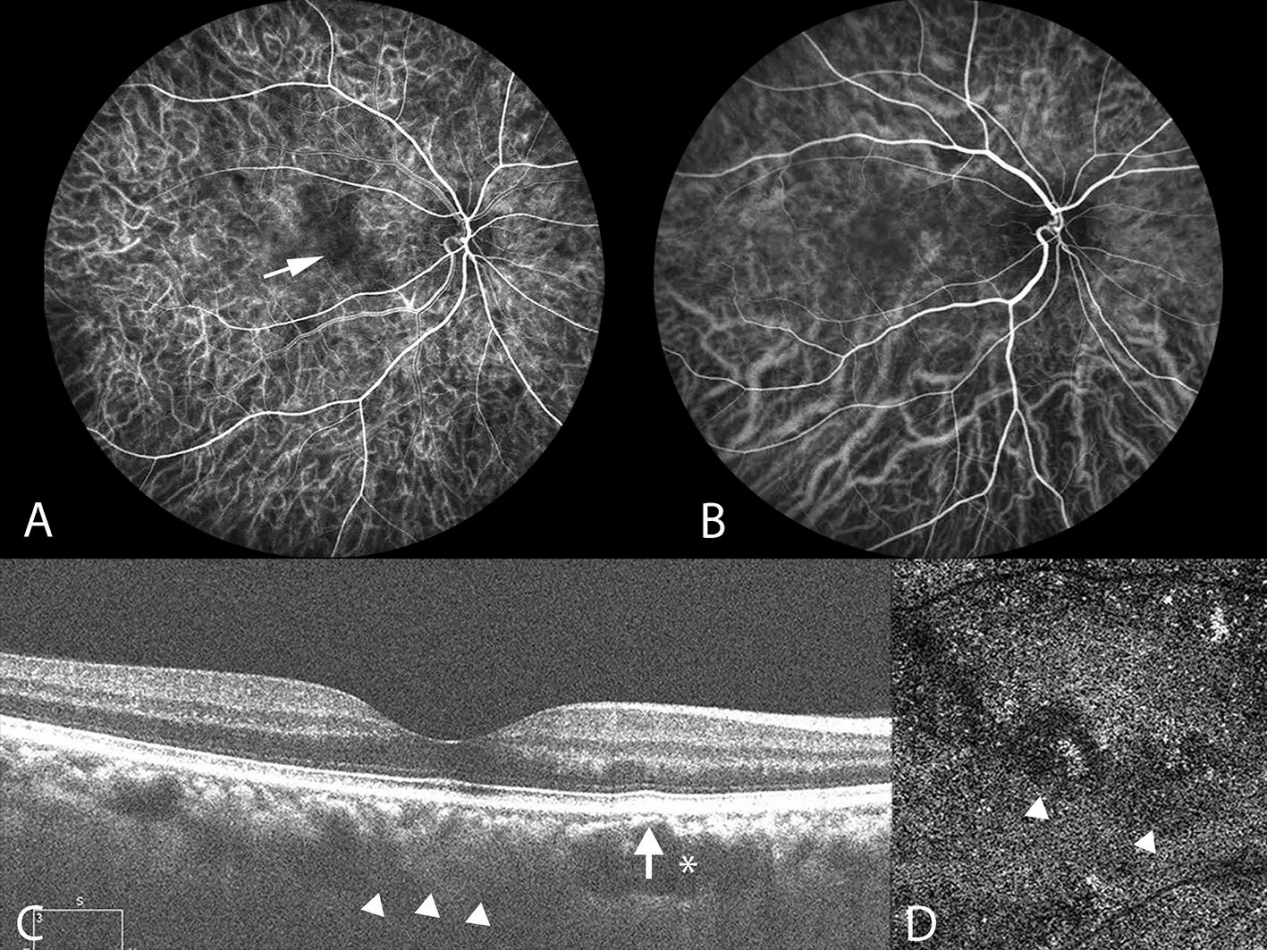
Multimodal imaging of the right eye of a 45-year-old man with chronic central serous chorioretinopathy. Pachyvessels and attenuation of the inner choroid are seen. (**A**) Indocyanine green video angiography image in the early phase revealing expanded hypofluorescence (white arrow), indicating a hypoperfused capillary segment. ICGA images revealing that the expanded hypofluorescence was colocalized with underlying pachyvessels (**B**) and choriocapillaris attenuation (**D**). (**B**) ICGA image indicating dilated choroidal vessels near the macula. Pachyvessels are shown as dilated choroidal vessels that do not taper toward the posterior pole. (**C**) Subretinal fluid resolved at the 4-month follow-up. Enhanced-depth imaging optical coherence tomography (OCT) demonstrated pachyvessels as larger hyporeflective lumen (white asterisk) and obliteration of overlying Sattler’s layer and choriocapillaris (white arrow). The inner surface of the sclera was demarcated, as indicated by the white arrowheads, and the subfoveal choroidal thickness was 315 μm. (**D**) Attenuation of flow signal within the choriocapillaris (white arrowheads) on OCT angiography.

Each of the senior ophthalmologists (CLJ, CWC, and TSH) diagnosed CSC by using multimodal images, including ICGA, FA, and SD-OCT images. Chronic CSC was defined as at least 3 months of symptoms, including metamorphopsia and blurred vision, with presence of subretinal fluid (SRF) located at the macula on SD-OCT images. Chronic CSC was characterized by RPE atrophy with mixed hyperautofluorescent and hypo-autofluorescent changes on fundus autofluorescence (FAF) images. Clinical features of pachyvessels were assessed using ICGA and enhanced-depth imaging OCT (EDI-OCT). On ICGA images, pachyvessels were differentiated from normal choroidal vessels as dilated choroidal vessels coursing through the posterior pole without tapering^5^ (Fig. 1B). Moreover, on EDI-OCT images, pachyvessels were observed as relatively large hyporeflective lumens (Fig. 1C). OCT angiography (OCTA; CIRRUS HD-OCT model 5000) was conducted on the choriocapillaris extending from 24 to 39 μm below the Bruch membrane. Manual segmentation was conducted to adjust the OCTA scans in order to obtain appropriate images. The subfoveal choroidal thickness (SFCT) was defined as the vertical distance from the outer border of the Bruch membrane to the inner surface of the sclera on EDI-OCT images (Fig. 1C). The measurement was taken below the fovea. At the initial visit, the baseline SFCT of CSC was measured by EDI-OCT. The BCVA of each patient was assessed at the initial visit and defined as the initial BCVA. After disease remission, the resolution of subretinal fluid on SD-OCT images and BCVA was assessed and recorded. CSC recurrence was defined as the presence of subretinal fluid on SD-OCT images or of angiographic leakage on FA images. Follow-up duration of CSC was defined as the period from the initial visit during which CSC diagnosis was established to the date of the last follow-up. The diagnostic criteria for PCV after CSC were based on EVEREST criteria^10^, including early focal hyperfluorescence on ICGA images and associated angiographic features. The time for PCV formation after CSC was defined as the period from CSC diagnosis on medical records to PCV diagnosis. The PCV morphology was characterized using ICGA^11^.

### Treatments for PCV

Treatments for PCV secondary to eyes with CSC included anti-VEGF monotherapy, PDT, or PDT and anti-VEGF combination treatment. Treatment for PCV was determined on the basis of visual acuity, lesion characteristics, and location. For anti-VEGF monotherapy, intravitreal injection at 4 weekly intervals with bevacizumab, ranibizumab, or aflibercept was chosen. For PDT, verteporfin (Visudyne; Novartis AG, Bulach, Switzerland) was infused at 6 mg/m^2^. Fifteen minutes after the start of infusion, a laser (light dose, 50 J/cm^2^; dose rate, 600 mW/cm^2^; wavelength, 689 nm) was applied to the entire PCV lesion for 83 s, as indicated by ICGA. For PDT and anti-VEGF combination treatment, anti-VEGF intravitreal injection was applied after PDT for 3 days.

### Statistical analysis

Continuous variables are presented as means ± standard deviations, and categorical variables are presented as frequencies and percentages. To compare continuous variables between the 2 groups, an independent samples *t* test was used for normally distributed variables, and the Mann–Whitney U test was used for nonnormally distributed variables. For categorical variables, the chi-square or Fisher exact test was used as appropriate. To determine the association between variables and the development of PCV in eyes with chronic CSC, logistic regression was conducted. Multivariate logistic regression was performed to minimize the effect of any potential confounders. *P* < 0.05 was considered statistically significant. All statistical analyses were performed using SPSS version 25.0 (SPSS Inc, Chicago, IL).

## Results

We reviewed 718 medical records with a discharge diagnosis of CSC for the period from 2009 to 2019. Among the 54 included patients with chronic CSC, 18 eyes of 18 patients developed PCV. Thirty-six eyes of 36 patients without evidence of PCV during follow-up were enrolled as the control group.

### Baseline characteristics

We included 54 eyes of 54 patients (47 men and 7 women) with chronic CSC. The mean age at the initial visit was 45.3 ± 9.8 years. The mean spherical equivalent refraction error was − 0.5 ± 1.7 diopters. The mean SFCT of CSC was 322.8 ± 119.5 μm at baseline. The average follow-up duration of CSC was 73.2 ± 42.9 months. Twenty patients (37.0%) had CSC recurrence. When patients initially presented with CSC, the mean BCVA logMAR (logarithm of the minimal angle of resolution) was 0.17 ± 0.21 in the PCV group and 0.10 ± 0.12 in the control group (*P* = 0.088); after the resolution of subretinal fluid on SD-OCT images, the mean BCVA logMAR was 0.09 ± 0.08 in the PCV group and 0.03 ± 0.06 in the control group (*P* = 0.001). Furthermore, 12 (66.7%) and 9 (25%) patients in the PCV and control groups, respectively, had pachyvessels on ICGA (*P* = 0.003). No between-group differences were observed in terms of baseline characteristics, including age, sex, refraction error, SFCT, CSC recurrence, or follow-up duration (Table 1).

**Table 1:** Baseline characteristics of patients with chronic CSC in this study. Values are presented as mean ± SD.

	Group		*P*
	without PCV formation (n = 36)	Subsequent PCV formation (n = 18)	
Male sex, no. (%)	32 (88.9%)	15 (83.3%)	0.674*
Age (years)	45.4 ± 10.8	44.9 ± 7.9	0.862^†^
Refractive error (S.E.)	− 0.3 ± 1.7	− 0.8 ± 1.8	0.796^‡^
LogMAR BCVA			
Initial	0.10 ± 0.12	0.17 ± 0.21	0.088^‡^
SRF resolved	0.03 ± 0.06	0.09 ± 0.08	0.001^‡^
SFCT (μm)	320.1 ± 92.4	325.3 ± 142.1	0.851^‡^
CSC recurrence, no. (%)	12 (33.3%)	8 (44.4%)	0.425**
Presence of pachyvessels, no. (%)	9 (25.0%)	12 (66.7%)	0.003**
Follow-up duration (months)	70.8 ± 39.7	77.9 ± 26.5	0.236^‡^

*P* value < 0.05 was considered statistically significant. *Fisher's exact test; ^†^independent *t* test; ^‡^Mann–Whitney test;

**Chi-square test; CSC, central serous chorioretinopathy; PCV, polypoidal choroidal vasculopathy; S.E., spherical equivalent; logMAR, logarithm of the minimal angle of resolution; SRF, subretinal fluid; SFCT, subfoveal choroidal thickness.

### Factors associated with PCV formation

Multivariate logistic regression analyses revealed that the presence of pachyvessels in chronic CSC was associated with PCV formation (odds ratio = 6.00; 95% CI, 1.74–20.68; *P* = 0.005) after adjustment for age and SFCT. PCV formation was not significantly associated with age, SFCT, or CSC recurrence. The results of multivariate logistic regression analyses for the assessment of clinical parameters are summarized in Table 2.

**Table 2:** The multivariate logistic regression for subsequent PCV Formation In Relation To Different Variables.

	OR	95% CI	*P* value*
Age	1.00	0.93–1.06	0.892
SFCT^†^	1.00	0.99–1.01	0.911
CSC recurrence	1.69	0.51–5.68	0.393
Presence of pachyvessels	6.00	1.74–20.68	0.005

*P value < 0.05 was considered statistically significant.

^†^Subfoveal choroidal thickness of CSC at baseline. OR, odds ratio; CI, confidence interval; PCV, polypoidal choroidal vasculopathy; CSC, central serous chorioretinopathy.

### Clinical features of PCV secondary to chronic CSC

Eighteen patients (15 men and 3 women) developed PCV after CSC. Their mean age of PCV diagnosis was 51 years (range 42–63 years). The mean (range) period from the diagnosis of CSC to evidence of PCV was 77.8 (27–108) months. The mean (range) SFCT of PCV was 327.7 (134–599) μm. On the basis of the morphology of PCV as determined on ICGA images, 10 patients (55.5%) had type 1 PCV (polypoidal CNV) and 8 (44.5%) had type 2 PCV (typical PCV). Treatments for PCV included anti-VEGF monotherapy (n = 9, 50%), PDT (n = 1, 5%), and PDT and anti-VEGF combination treatment (n = 4, 22.2%); 4 patients received no treatment. Among the 9 patients who received monthly anti-VEGF monotherapy, 5 had resolution of subretinal fluid on OCT images, 3 had persistent subretinal fluid on OCT images during follow-up, and 1 lost to follow-up. The mean (range) number of anti-VEGF injections was 4.6 (1.0–11.0). One patient received PDT monotherapy, and resolution of subretinal fluid on OCT images was noted. Four patients received PDT and anti-VEGF combination treatment and all of the 4 patients had resolution of subretinal fluid on OCT images. The clinical features of PCV secondary to chronic CSC are summarized in Table 3.

**Table 3:** Clinical Features of PCV Subsequent to Chronic CSC. *Lost of follow-up; F, female; M, male; SFCT, subfoveal choroidal thickness; SRF, subretinal fluid; PDT: photodynamic therapy; VEGF, vascular endothelial growth factor; PCV, polypoidal choroidal vasculopathy.

Case	Age	Gender	Time to PCV formation (months)	SFCT (μm)	PCV morphology	Treatment	Response
1	50	F	104	306	PCV type 1	Anti-VEGF	Resolution
2	63	M	45	163	PCV type 2	Anti-VEGF	SRF persisted
3	46	M	84	429	PCV type 1	Anti-VEGF	N/A*
4	63	F	27	339	PCV type 2	Anti-VEGF	Resolution
5	54	M	48	318	PCV type 1	Anti-VEGF	Resolution
6	52	M	62	355	PCV type 1	–	
7	47	F	105	287	PCV type 1	Anti-VEGF	SRF persisted
8	46	M	92	259	PCV type 1	–	
9	42	M	32	246	PCV type 1	–	
10	57	M	108	516	PCV type 1	Anti-VEGF	Resolution
11	42	M	65	589	PCV type 2	Anti-VEGF	SRF persisted
12	53	M	90	599	PCV type 1	PDT	Resolution
13	49	M	72	134	PCV type 1	Anti-VEGF	Resolution
14	42	M	98	237	PCV type 2	PDT + Anti-VEGF	Resolution
15	52	M	106	159	PCV type 2	PDT + Anti-VEGF	Resolution
16	47	M	74	184	PCV type 2	PDT + Anti-VEGF	Resolution
17	62	M	82	464	PCV type 2	PDT + Anti-VEGF	Resolution
18	51	M	108	315	PCV type 2	–	

## Discussion

In this retrospective observational study involving chronic CSC, the clinical features of pachyvessels are associated with the subsequent development of PCV. CSC recurrence and different SFCT of chronic CSC are not associated with subsequent PCV formation. The PCV subsequent to chronic CSC is characterized by a slightly younger age group.

**Figure 2:**
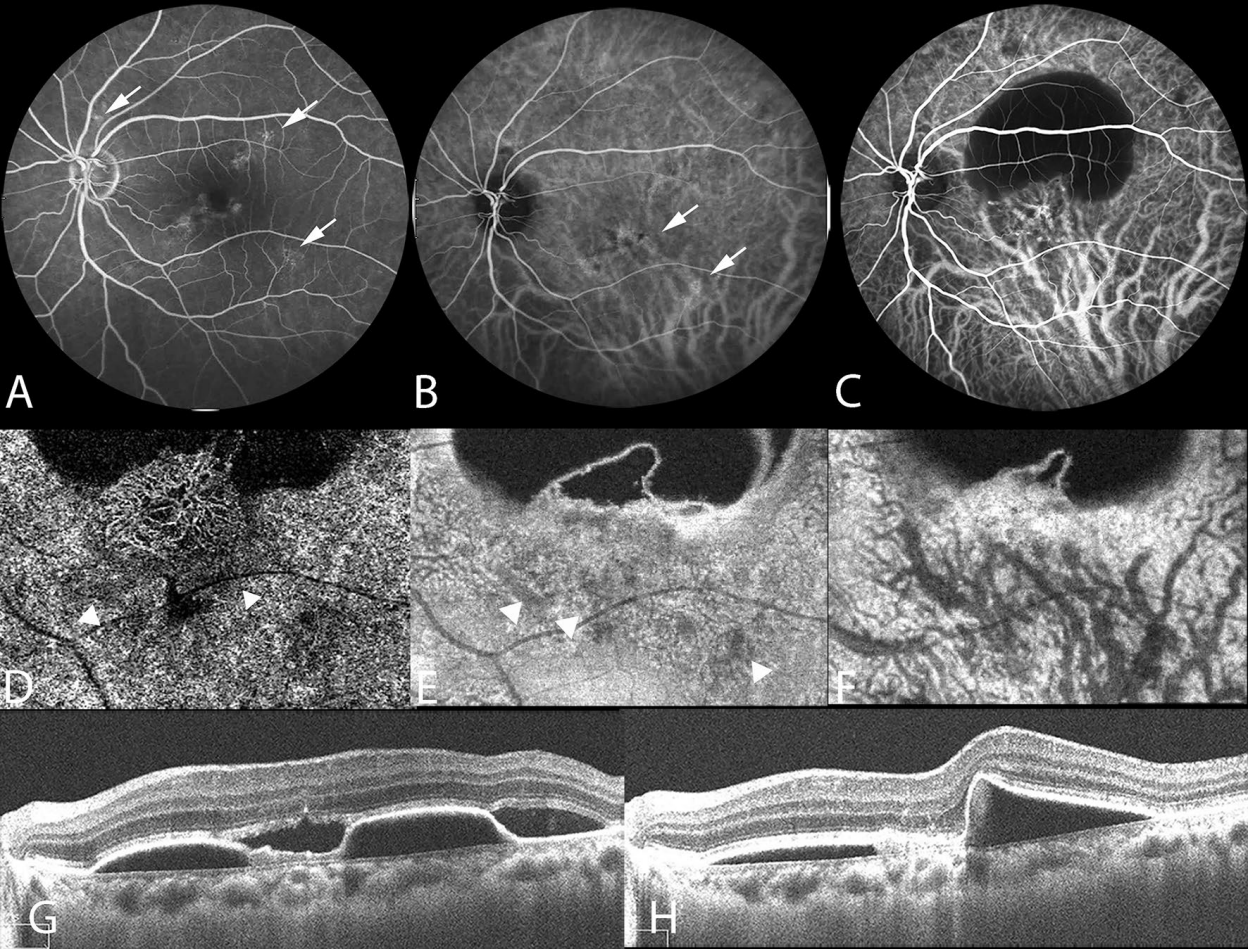
Multimodal imaging of the left eye of a 38-year-old woman with chronic central serous chorioretinopathy and subsequent polypoidal choroidal vasculopathy. (**A**) Fluorescein angiography revealing focal leakage points (white arrows). (**B**) Multifocal hyperfluorescent areas (white arrows) shown on the indocyanine green angiography (ICGA) in the middle phase. Pachyvessels can be seen in the inferotemporal quadrant; they do not taper toward the posterior pole and terminate abruptly. (**C–H**) Polypoidal choroidal vasculopathy developed subsequently at the 10-year follow-up. (**C**) ICGA revealing a branching vascular network with terminal aneurysmal dilatations. (**D**) Optical coherence tomography angiography (OCTA) image, with segmentation from 19 to 49 μm with respect to the retinal pigment epithelium (RPE), demonstrating type 1 (sub-RPE) neovascularization overlaying the attenuated choriocapillaris (white arrowheads). (**E**) Focal inward displacement of the underlying pachyvessels (white arrowheads) on OCTA image, with segmentation from 24 to 44 μm with respect to Bruch’s membrane. (**F**) OCTA image, with segmentation from 64 μm to 125 μm with respect to Bruch’s membrane, revealing dilated choroidal vessels. (**G**) OCT image revealing an elevated RPE detachment and the presence of subretinal fluid. (**H**) OCT image showing resolution of subretinal fluid following two doses of monthly intravitreal injection of anti-VEGF.

In this study, the presence of pachyvessels in CSC is associated with morphological alterations of choroidal structure and the presence of sub-RPE neovascularization. ICGA is crucial for the evaluation of morphological changes of choroidal vessels^11,12^. Our study demonstrated that the hypoperfused area determined on ICGA images, corresponding to choriocapillaris signal attenuation on OCTA images, was correlated with the presence of underlying pachyvessels (Fig. 1). A previous study revealed that the area of flow impairment in the choriocapillaris layer, as determined on OCTA images, was increased in the PPE group compared with that in the control group, and pachyvessels underlay 89.0% of the signal void area^13^. Furthermore, our follow-up images revealed that pachyvessels were colocalized with expanded ischemic areas of the choriocapillaris, which were subsequently overlay by sub-RPE neovascularization (Fig. 2). Thus, our data supports that pachyvessels are associated with PCV formation in eyes with chronic CSC.

The SFCT is a widely used parameter to identify pachychoroid diseases, and its value is influenced by factors such as age, axial length, blood pressure, and time of the day^5,14^. Previous investigations have proposed that SFCT > 300 μm can be considered to indicate a pathological condition^5,15^. However, Spaide and Ledesma-Gil demonstrated that no difference was noted for choriocapillaris flow deficits between a pachychoroid group without disease and normal controls^16^. Based on analyses in this study, SFCT of chronic CSC is not associated with subsequent development of PCV. Our EDI-OCT findings indicate that structural changes in the choriocapillaris were not correlated with the SFCT, supporting the concept that pachychoroid is not necessarily pathologic.

Long-term follow-up of eyes with CSC indicated a recurrence rate of 30% to 50.7%^1^. CSC recurrence was not associated with PCV formation, according to our results. However, follow-up imaging of eyes with CSC recurrence indicated an expanded area of diseased RPEs on FAF images and flat-irregular PEDs on OCT images. Complete multimodal image examinations might help detect neovascular formations in eyes with recurrent CSC^12^. With respect to the between-group difference of BCVA after resolution of subretinal fluid, our results reveal that BCVA was worse in the PCV group than in the control group, which might be related to the interruption of the ellipsoid zone and more diseased RPEs, as observed on FAF images, due to a longer period of fluid accumulation. Previous research indicated similar results^1^, demonstrating the necessity of timely intervention for recurrent and sustained fluid accumulation.

The prevalence of PCV is variable among ethnicities, and PCV is commonly diagnosed in patients aged in their late fifties to seventies^17,18^. By contrast, PCV after chronic CSC is commonly diagnosed in patients aged in their early fifties, on average; this may suggest that the etiology of such PCV differs from that of neovascular AMD. In addition, 66.7% of patients with PCV after CSC had pachyvessels, and their average SFCT was 327.7 μm, which is consistent with previous findings of large choroidal thicknesses in PCV^18^. A previous study including > 300 eyes with PCV indicated that the SFCT exhibited a bimodal distribution, with peaks at 170 and 390 μm^19^. These findings indicate that this phenotype of PCV has similar clinical features to the pachychoroid disease spectrum, although the pathogenesis remains unclear.

Both PDT and anti-VEGF treatments appear to have important roles in the treatment of PCV after chronic CSC. Among the patients who received PDT monotherapy or PDT and anti-VEGF combination treatment, all of them had disease remission. We also found that 60% of the patients had polypoidal lesion regression on ICGA images at the 3-month follow-up. However, a previous study had suggested that recurrent exudation was common in up to 50–60% of eyes following PDT monotherapy for PCV^5^. The long-term effect of PDT therapy for PCV after chronic CSC should be evaluated in the future. One-third of the patients who received monthly anti-VEGF therapy had persistent subretinal fluid on OCT images. Furthermore, the sub-RPE neovascularization showed continued growth, forming new lesions over time. A classification of PCV based on ICGA imaging proposed that the type 2 PCV had pathological polypoidal lesions as result of hyalinized arteriosclerosis and vascular wall alterations^11^. This pathological features might be related to poor response of PCV to anti-VEGF treatment. Another study suggested that the pachychoroid subtype of PCV had a poor response to anti-VEGF monotherapy^20^.

The treatment of PCV with different choroidal thicknesses has yet to be determined^7,20^. In the EVEREST-II study, anti-VEGF and PDT combination therapy achieved superior outcomes than anti-VEGF monotherapy in terms of BCVA gain and polyp closure at 2 years^21^. Patients with PCV with relatively large SFCTs exhibited a favorable improvement in visual acuity when subjected to PDT and anti-VEGF combination treatment^18^, which is consistent with the findings of the present study. Currently, intravitreal anti-VEGF therapy with or without adjunctive PDT is commonly used to treat PCV. Results of the PLANET studies revealed that anti-VEGF monotherapy and anti-VEGF and PDT combination therapy could achieve equivalent visual outcomes at 2 years^22^. Accordingly, anti-VEGF monotherapy may be a solution for areas without adequate access to PDT. Decisions on the treatment for PCV related to the pachychoroid disease spectrum should consider multiple factors, including visual acuity, lesion characteristics, location, and individual patients and settings. Large-scale longitudinal studies are warranted to determine the treatment direction more definitively.

**Figure 3:**
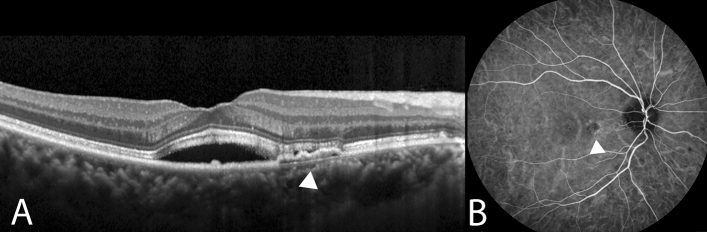
Multimodal imaging of the right eye of a 62-year-old woman with pachychoroid neovasculopathy and with a history of chronic central serous chorioretinopathy. (**A**) Enhanced-depth imaging optical coherence tomography (OCT) demonstrated shallow irregular RPE detachment (double-layer sign) (white arrowhead) with subretinal fluid. (**B**) Choroidal hyperpermeability was indicated as hyperfluorescence (white arrowhead) on the indocyanine green angiography (ICGA) in the early-mid phase.

The pathogenetic mechanisms underlying pachychoroid diseases, including CSC, PNV, and PCV, involve structural alterations of the choroid and exudative changes^6^. Choroidal vascular hyperpermeability on ICGA images may imply relative ischemia engendered by damage to the choriocapillaris. PCV with features of choroidal vascular hyperpermeability was reported to be associated with a history of CSC^23^. Patients with PCV with choroidal vascular hyperpermeability were associated with the G allele of *age-related maculopathy susceptibility 2 (ARMS2)* A69S and the T allele of *complement factor H (CFH)*, which are risk alleles for CSC^24^. Pachychoroid neovasculopathy is a type 1 neovascularization overlying focal areas of choroidal thickening and dilated choroidal vessels^25^. Shallow irregular RPE detachment on OCT images and absence of branching vascular network with aneurysmal lesions on ICGA images distinguish PNV from PCV (Fig. 3). A previous study reported that cases with PNV subsequently developed polypoidal structures^4^. Though the pathogenesis remains unclear, PNV and PCV may represent different stages of the same spectrum disease, and future investigations on the association between these diseases will help guide treatment.

This study is limited by its retrospective design and small sample size. When patients with CSC lost to follow-up after disease remission, it was difficult to clarify the temporal sequence. In addition, we established stringent inclusion criteria to minimize potential confounding factors. Our clinical data revealed the pachyvessel morphology and choriocapillaris flow deficits in a portion of patients with chronic CSC, who tended to receive laser treatment or PDT because of impaired vision. Because laser treatment and PDT alter the choriocapillaris^26^, patients who had received these treatments were excluded from this study. Pachyvessels were associated with overlying choriocapillaris flow attenuation. Various protocols for the assessment of the choriocapillaris have been proposed^16,27^. Future studies should quantitatively evaluate changes in the choriocapillaris as result of pachyvessel morphology to elucidate the relationship between the choroidal structure and sub-RPE neovascularization.

In conclusion, our results suggest that the clinical features of pachyvessels in chronic CSC are associated with the subsequent development of PCV. This PCV phenotype is characterized by a slightly younger age group. The presence of pachyvessels in patients with chronic CSC may be a risk factor for neovascular formation; accordingly, clinicians should consider this when evaluating patients with CSC.

## Data Availability

The datasets generated during and/or analysed during the current study are available from the corresponding author on reasonable request.
